# Editorial: Case reports in thrombosis: 2023

**DOI:** 10.3389/fcvm.2024.1446992

**Published:** 2024-07-01

**Authors:** Luca Spiezia, Nicholas Kipshidze

**Affiliations:** ^1^ First Chair of Internal Medicine, Department of Medicine, Padova University Hospital School of Medicine, Padova, Italy; ^2^ Icahn School of Medicine & The Mount Sinai Hospital, New York, NY, United States

**Keywords:** hickman catheter, acute pulmonary embolism, atrioventricular block, cardiac transthyretin amyloidosis, extracorporeal membrane oxygenation

**Editorial on the Research Topic** Case reports in thrombosis: 2023

This editorial presents a collection of articles published in Frontiers in Cardiovascular Medicine: Case Reports in Thrombosis: 2023. This year as well, we have selected a wide range of case reports which, in the field of thrombotic pathology, deal with both basic research and clinical topics, from diagnosis to therapy. We hope that readers will appreciate our effort which has focused on proposing current papers, written with a solid and rigorous methodology, and of great interest to the scientific community at large. In this regard, our thanks go to all the authors who, by submitting their manuscripts for this special edition, made it possible; and to all the reviewers who with dedication and competence contributed to completing a product that we believe is of the best quality. Finally, we would like to thank the editorial office for their excellent work both in support of the authors and ourselves, the co-topic Editors.

The following are the articles in the Frontiers in Cardiovascular Medicine: Case Reports in Thrombosis: 2023.

*Case report: Transected Hickman catheter and its thrombotic occlusion in a patient with idiopathic pulmonary arterial hypertension—can a catheter replacement be avoided?, by Grzegorz Sławiński et al.*

Catheter-related thromboses are increasingly frequent complications in clinical practice that can have serious consequences for the patient (1). In fact, when thrombosis occludes the catheter, it is necessary to proceed with the removal of the catheter and its repositioning. This case report describes a mechanical technique to restore the patency of the lumen of the catheter, thus avoiding its removal*.* The validation of this novel technique would allow to avoid the possibility of vascular damage associated with repeated removal and repositioning of catheters.

*Acute pulmonary embolism combined with acute myocardial infarction as the first manifestation of acute leukemia: a case report, by Shuzhan Zheng et al.*

It is well known that both solid and hematological malignancies are associated with an increase in both thrombotic and haemorrhagic risk (2). The peculiarity of this case report lies in the fact that the thrombotic event — and specifically in this case simultaneous pulmonary embolism and acute myocardial infarction — preceded the diagnosis of neoplasia. Therefore, it is always important to remember that the occurrence of a thrombotic event may be the first manifestation of an occult cancer.

*Case Report: PROS1 (p.Leu584Arg) pathogenic mutation causes portal and superior mesenteric venous thromboembolism, by Peng Ding et al.*

Several cases of thrombosis and particular unusual-site thrombosis are categorized as unprovoked, meaning that clinicians are unable to pinpoint the underlying risk factor and etiopathogenesis. It is very likely that a substantial proportion of these patients may be carriers of an unknown hereditary thrombophilia which may explain the thrombotic event as is the case in classic hereditary thrombophilia (3). The identification of novel genetic mutations associated with thrombophilias will help better characterize patients with thrombosis of unknown origin.

*Case report: One case of umbilical vein thrombosis in the second trimester with associated portal vein thrombosis after childbirth, by Wei-Wei Dai et al.*

The authors described a rare case of antenatal diagnosis of umbilical vein thrombosis. An ultrasound performed soon after birth identified the presence of portal vein thrombosis. Although rare, thrombosis of the umbilical vein is a very alarming complication whose diagnosis remains clinically challenging and which requires further studies to better understand the mechanisms and possible risk factors.

*Case Report: Successful conversion and salvage resection of huge hepatocellular carcinoma with portal vein tumor thrombosis and intrahepatic metastasis* via *sequential hepatic arterial infusion chemotherapy, lenvatinib plus PD-1 antibody followed by simultaneous transcatheter arterial chemoembolization, and portal vein embolization, by Xin Luo et al.*

Hepatocellular carcinoma is a neoplastic disease that is often complicated by thrombotic events (4). When, as described in this case report, the disease is too advanced and major hepatectomy is not feasible, developing sequential loco-regional techniques that can reduce the neoplastic mass and make it more manageable surgically treatment can become a lifesaving measure.

*Case Report: Complete atrioventricular block in an elderly patient with acute pulmonary embolism, by Moojun Kim et al.*

This case report describes a peculiar case of complete atrioventricular block due to acute pulmonary embolism. It bears highlighting that the patient first underwent a catheter-directed thrombolysis immediately followed by a temporary pacemaker insertion, which was later removed after stable sinus rhythm recovery.

*Cryptogenic ischemic stroke in cardiac transthyretin amyloidosis and sinus rhythm: a case report, by Angela Napolitano et al.*

Transthyretin amyloidosis is known to be associated with a significant increase in thrombotic risk, though the underlying mechanisms are still largely unknown (5). It is therefore critical to study the coagulation profile of these patients using non-traditional methods, such as thromboelastometry, thrombin generation, and/or microparticle measurement, in order to better understand the mechanisms of hypercoagulability and identify patients at higher prothrombotic risk.

*ECMO management for severe pulmonary embolism with concurrent cerebral hemorrhage: a case report, by Lutao Xie et al.*

Cardiac arrest due to acute pulmonary embolism is fortunately a rare occurrence, though it may turn fatal if/when all conventional resuscitation maneuvers fail (6). In this case report, the authors describe how veno-arterial extracorporeal membrane oxygenation (ECMO) may be a life-support tool, as well as how to manage the anticoagulant treatment during ECMO in case of concomitant cerebral hemorrhage.
